# An optimal protective ventilation strategy in lung resection surgery: a prospective, single-center, three-arm randomized controlled trial

**DOI:** 10.1007/s13304-025-02091-7

**Published:** 2025-01-21

**Authors:** Seihee Min, Susie Yoon, Hyun Woo Choe, Haesun Jung, Jeong-Hwa Seo, Jae-Hyon Bahk

**Affiliations:** 1 Department of Anesthesiology and Pain Medicine, Chung-Ang University Gwangmyeong Hospital, Chung-Ang University College of Medicine, 110 Deokan-ro, Gwangmyeong-si, Gyeonggi-do 14353 Republic of Korea; 2 Department of Anesthesiology and Pain Medicine, Seoul National University Hospital, Seoul National University College of Medicine, 101 Daehak-ro, Jongno-gu, Seoul, 03080 Republic of Korea

**Keywords:** Protective ventilation, One-lung ventilation, Thoracic surgery, Postoperative pulmonary complications

## Abstract

Protective ventilation reduces ventilator-induced acute lung injury postoperatively; however, the optimal strategy for one-lung ventilation (OLV) remains unclear. This study compared three protective ventilation strategies with a postoperative partial pressure of oxygen (PaO_2_)/fraction of inspired oxygen (FiO_2_) ratio to reduce the incidence of immediate postoperative pulmonary complications (PPCs) in patients undergoing lung resection surgery. Eighty-seven patients with ASA physical status I–III requiring OLV for lung resection surgery were randomized into three groups according to the applied ventilation strategies: low tidal volume (V_T_) of 4 mL/kg of predicted body weight (PBW) (LV group), medium V_T_ of 6 mL/kg of PBW (MV group), and high V_T_ of 8 mL/kg of PBW (HV group). All patients received 5 cmH_2_O of positive end-expiratory pressure (PEEP). The primary outcome was the mean difference of PaO_2_/FiO_2_ ratio after surgery. The radiologic findings of acute lung injuries were also evaluated. The incidence of immediate PPCs was determined by PaO_2_/FiO_2_ ratio of < 300 mmHg and/or newly developed radiological findings within 72 h after surgery. The MV group showed the highest PaO_2_/FiO_2_ ratio at 6 h postoperatively (*P* = 0.010). There were no significant among-group differences in radiological findings in 3 postoperative days. The MV group showed the lowest incidence of immediate PPCs among the three groups (*P* = 0.007). During OLV in lung resection surgery, protective ventilation at a V_T_ of 6 mL/kg with PEEP of 5 cmH_2_O may achieve a higher postoperative PaO_2_/FiO_2_ ratio, reducing the incidence of immediate PPCs.

## Introduction

Postoperative pulmonary complications (PPCs) are common and seriously morbid, especially after thoracic surgery [1, 2]. Ventilator-induced lung injury (VILI) triggers postoperative acute lung injury (ALI), which is caused by volutrauma, barotrauma, atelectrauma, and oxygen toxicity during mechanical ventilation [3, 4].

A protective ventilation strategy has been developed to address VILI. This strategy involves a low tidal volume (V_T_), a certain amount of positive end-expiratory pressure (PEEP), a low fraction of inspired oxygen (FiO_2_), and alveolar recruitment [5–7]. Although this strategy reduces PPCs, applying protective ventilation during one-lung ventilation (OLV) is challenging given the required appropriate compromise between hypoxemia and ALI.

Several observational studies, randomized-controlled trials, and meta-analyses have attempted to establish the appropriate ventilatory settings during OLV [3, 8–16]. A recent meta-analysis of randomized-controlled trials reported that a VT of 4–7 mL/kg with PEEP of 5–10 cmH_2_O were superior to V_T_ of 8–15 mL/kg with PEEP of 0–5 cmH_2_O [14]. However, a large observational study on 29,000 patients showed that a V_T_ of 6 mL/kg was a risk factor for PPCs [15]. Additionally, a post-hoc prospective multicenter study investigated PPCs in patients with low V_T_ (7.4 ± 1.6 mL/kg) with a PEEP of 3.5 ± 2.4 cmH_2_O [16]. Taken together, although there is an established consensus regarding the adoption of a low V_T_ with a certain amount of PEEP, the appropriate conditions for V_T_ remain unclear for OLV.

Therefore, this three-arm comparison study aimed to determine the optimal protective ventilation strategy for OLV that is safe and beneficial for postoperative oxygenation, resulting reduced risks of immediate PPCs after lung resection surgery.

## Methods

### Ethical approval

This study was approved by the Institutional Review Board and registered at http://clinicaltrials.gov. This study was conducted according to the Good Clinical Practice guidelines and principles of the Declaration of Helsinki, as revised in 2013. Written informed consent was obtained from all the participants.

### Study population and randomization

The inclusion criteria included age > 18 years, an American Society of Anesthesiologists physical status of I–III, and requiring OLV for elective lung resection surgery. Patients with previous lung resection, baseline partial pressure of oxygen (PaO_2_) < 70 mmHg, preoperative supplemental oxygen treatment, tracheostomy, or chronic obstructive pulmonary disease with forced expiratory volume in 1 s over a forced vital capacity < 70% were excluded. Each patient underwent preoperative arterial blood gas analysis (ABGA), pulmonary function tests, and chest radiography.

The patients were randomly allocated to three groups according to OLV strategies as follows: those ventilated at 4 mL/kg of predicted body weight (PBW) (n = 29, low V_T_ [LV] group), those ventilated at 6 mL/kg of PBW (n = 29, medium V_T_ [MV] group), and those ventilated at 8 mL/kg of PBW (n = 29, HV group). PEEP of 5 cmH_2_O was applied to all patients.

Randomization was conducted using a computer-generated program (http://www.randomization.com) at a 1:1:1 allocation ratio. An anesthetic nurse not involved in the study generated a random allocation sequence using sealed opaque envelopes. The patients, investigators, surgeons, and data collectors were blinded to the group allocation. Only the bedside anesthesiologists who regulated the ventilator and maintained intraoperative anesthesia were not blinded.

### Anesthetic management

All the patients were monitored using 3-lead electrocardiography, noninvasive arterial blood pressure measurement, peripheral oxygen saturation (SpO_2_), bispectral index, and acceleromyography. General anesthesia was induced and maintained with intravenous propofol and remifentanil using an effect-site target-controlled infusion pump (Module DPS Orchestra®, Brezins, France). Propofol was adjusted to 3–5 µg/mL, while remifentanil was adjusted to 2–6 ng/mL according to the effect-site concentrations.

After confirming the patients’ loss of consciousness, a double-lumen tube (DLT) was intubated after injecting 0.6 mg/kg of rocuronium. When the bispectral index was < 60 and the train-of-four (TOF) count was 0, the patients were intubated with a DLT (Mallinckrodt endobronchial tube; Covidien, Mansfield, MA, USA) using direct or video laryngoscopy. After confirming the appropriate position of the DLT using a fiberoptic bronchoscope (FOB) (LF-GP; Olympus Optical Co., Tokyo, Japan), the tracheal and bronchial cuffs were inflated at a pressure of 20–25 cmH_2_O using a capnometer for lung isolation.

During the surgery, 0.2–0.3 mg/kg of rocuronium was intermittently administered for muscle relaxation. The anesthetic depth was adjusted to achieve a target bispectral index of 40–60. Lactated Ringer’s solution was infused at a rate of 3–5 mL/kg/h as maintenance fluid.

### Study protocol

The radial artery was catheterized to monitor continuous arterial blood pressure and for intraoperative blood sampling. During arterial and central line catheterization, both lungs were ventilated at a V_T_ of 10 mL/kg of PBW with a PEEP of 5 cmH_2_O and an inspiratory to expiratory ratio of 1:2 with a fresh gas flow of 2 L/min in FiO_2_ 1.0 using a mechanical ventilator (Primus; Dräger, Lübeck, Germany). End-tidal carbon dioxide (EtCO_2_) or arterial tension of carbon dioxide (PaCO_2_) was maintained at 35–45 mmHg by adjusting the ventilatory respiratory rate (RR).

After the patient was placed in the lateral decubitus position and the final position of the DLT was confirmed using an FOB for the operation, OLV was initiated at a V_T_ of 4, 6, or 8 mL/kg of PBW according to assigned group, while other ventilatory settings were maintained. FiO_2_ was downregulated to 0.8 after alveolar recruitment with an inspiratory pressure of 20 cmH_2_O for 15–20 s. During OLV, FiO_2_ was reduced stepwise from 0.8 to 0.5 if the patients did not show hypoxemia, which was defined as a decrease in the arterial tension of oxygen (PaO_2_) to < 80 mmHg or SpO_2_ to < 95%. Each FiO_2_ level was maintained for 5 min. In patients showing hypoxemia during OLV, FiO_2_ was increased stepwise to 1.0. If hypoxemia could not be corrected by increasing the FiO_2_ to 1.0, we considered endobronchial suctioning followed by alveolar recruitment, reassessing the DLT position with an FOB, applying continuous positive airway pressure at 2 cmH_2_O, and applying intermittent two-lung ventilation (TLV) as rescue interventions. The rescue interventions were recorded.

Additionally, peak inspiratory pressure (PIP) was monitored to ensure it did not exceed 35 cmH_2_O during mechanical ventilation. If the PIP was > 35 cmH_2_O during OLV, we provided endobronchial suctioning followed by alveolar recruitment and reassessed the DLT position.

After lung resection, TLV was resumed after gentle suctioning of both lungs, followed by alveolar recruitment to reexpand the operated lung. The same ventilatory setting as applied before OLV was implemented, except for an FiO_2_ of 0.4 until the surgery was completed. Patients who underwent open thoracotomy due to unexpected adhesion or received blood transfusion due to an estimated blood loss > 500 mL intraoperatively were also recorded.

After surgery completion, intravenous or epidural patient-controlled analgesia comprising fentanyl, morphine, and ramosetron were administered according to the surgical technique. After placing the patients in the supine position, oral and bronchial secretions were removed. The DLT was extubated after the administration of 2–4 mg/kg sugammadex and the patient showed recovery of adequate spontaneous breathing, reflex, and a TOF ratio of > 0.9.

Patients were transferred to postanesthetic care unit (PACU) or surgical intensive care unit (SICU) at the surgeons’ discretion with consideration of the surgical procedure, intraoperative surgical events, or requirement of close monitoring and appropriate postoperative care according to patient’s underlying disease. During their transfer, the patients wore a Venturi mask (MOW Medical, Wonju, Korea) at FiO_2_ of 0.4 to ensure consistent FiO_2_ delivery. Supplemental oxygen was changed stepwise by the surgeon to maintain adequate oxygen saturation; if oxygen supply was not required, the patients were ventilated on room air.

### Measurement of outcomes

The primary outcome was the mean difference in PaO_2_/FiO_2_ ratio among the three groups after surgery. Postoperative ABGA was performed by an investigator immediately after the patients arrived at the PACU or SICU and 6 h after the surgery. The PaO_2_/FiO_2_ ratio was calculated in terms of PaO_2_ values and the supplied oxygen at the measurement time points in ABGA. Chest radiographs were obtained each morning for 3 postoperative days. A chest radiologist blinded to the study analyzed the radiographs to evaluate newly developed lung lesions, including lung infiltrations and atelectasis. The incidence of immediate PPCs, which were represented by acute lung injuries determined by a PaO_2_/FiO_2_ ratio of < 300 mmHg and/or radiological findings within 72 h after surgery, was also compared among the groups.

Secondary outcomes included hemodynamic variables; intraoperative ABGA; PaO_2_/FiO_2_; and ventilator settings, including V_T_, RR, FiO_2_, mean airway pressure (P_mean_), airway plateau pressure (P_plat_), and PIP recorded at five consecutive time points: T_TLV_baseline_, after anesthesia induction; T_OLV_20min_, 20 min after OLV; T_OLV_40min_, 40 min after OLV; T_OLV_60min_, 60 min after OLV; and T_TLV_20min_, 20 min after TLV resumption. PaO_2_/FiO_2_ was calculated according to PaO_2_ and applied FiO_2_ via a ventilator at the measurement time point.

Additional intraoperative treatment options for hypoxemia, rescue interventions, and respiratory and cardiovascular events were also recorded. Moreover, individual postoperative pain was assessed using a visual analog scale at 30 min after arriving in the PACU or SICU. Additionally, data regarding the length of stay in the PACU or SICU, hospitalization, readmission to the ICU due to PPCs, and mortality at 30 postoperative days were recorded. Data were assessed by an investigator who was not involved in the patient’s anesthetic care or postoperative management.

### Statistical analyses

The sample size was calculated based on previously published data showing a difference in PaO_2_/FiO_2_ ratios measured 15 min after resuming TLV at the end of the surgery with the three ventilation strategies [17]. An a priori power analysis of the previous data indicated that 24 patients were required in each group, with an estimated effect size of 0.796 (α error of 0.05) and a power of 90%. Accounting for a dropout rate of 20%, the final sample size was 29 patients per group. Statistical analyses were performed using G Power software (version 3.1, University of Düsseldorf, Germany).

The R language version 4.1.2 (R Foundation for Statistical Computing, Vienna, Austria) and T&F program version 4.0 (YooJinBioSoft, Korea) were used for all analyses. Continuous variables are expressed as medians (interquartile ranges). The sample numbers and percentages were computed for categorical variables.

The mean differences among the three groups were analyzed using the Kruskal–Wallis *H* test. The chi-squared test with continuity correction or Fisher’s exact test was performed to analyze the proportion of sample numbers in the subgroups of categorical variables. The Bonferroni method was used for correction of multiple comparisons. *P* < 0.05 was considered statistically significant. All data were analyzed according to the intention-to-treat principle.

## Results

### Characteristics of patients, surgery, and anesthesia

**Fig. 1:**
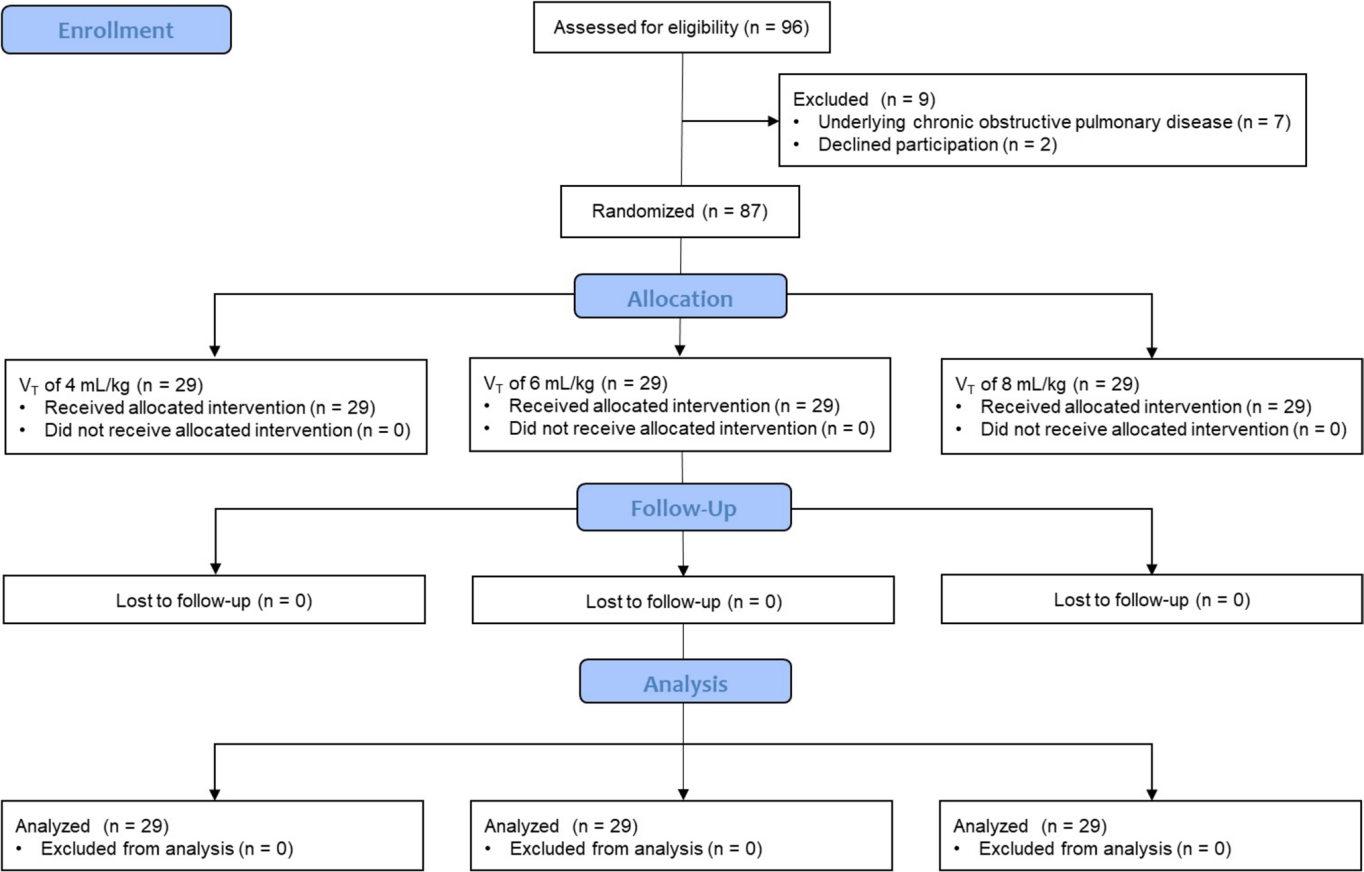
CONSORT diagram. *V*_*T*_ tidal volume, *PIP* peak inspiratory pressure, *TLV* two-lung ventilation, *CONSORT* Consolidated Standards of Reporting Trials

Among 96 eligible patients, 87 were enrolled and randomized into three groups (n = 29 each) from July 2017 to January 2018. Since no patients were excluded, 29 patients in each group were analyzed according to intention-to-treat analysis (Fig. 1).

Table 1 shows the patient characteristics and intraoperative variables. There were no differences in the demographic or intraoperative data among the groups. Upon completion of surgery, all patients were transferred to the PACU or SICU after tracheal extubation, and no patient was reintubated due to respiratory distress in the recovery room.

**Table 1:** Characteristics of the patients, surgery, and anesthesia

Variable	LV group (n = 29)	MV group (n = 29)	HV group (n = 29)	*P* value
Age (years)	62 (58–70)	64 (55–70)	58 (52–67)	0.335
Female	15	14	13	0.963
Height (cm)	163 (156–170)	165 (156–171)	164 (157–169)	0.851
Weight (kg)	59 (55–68)	59 (52–69)	62 (56–71)	0.392
Predicted body weight (kg)	56 (50–65)	59 (49–67)	60 (50–64)	0.960
Body mass index (kg/m^2^)	23.2 (21.1–24.7)	23.1 (20.5–25.8)	24.9 (22.3–26.8)	0.097
ASA physical status (I/II)	7/22	11/14	9/20	
Underlying disease				
(hypertension/diabetes/stroke/heart disease)	7/5/0/8	11/3/1/3	6/1/1/3	
Pulmonary function test				
FEV_1_/FVC (% predicted)	77 (74–80)	80 (75–85)	78 (74–83)	0.111
Type of operation				
(wedge resection/segmentectomy/lobectomy)	3/4/19	4/2/23	3/5/19	
VATS/Open thoracotomy	25/4	29/0	26/3	
Amount of anesthetic drugs and fluids				
Propofol (mg)	1100 (908–1566)	1200 (901–1654)	1280 (1054–1596)	0.478
Remifentanil (μg)	1143 (965–1510)	1200 (896–1504)	1500 (1000–1749)	0.311
Rocuronium (mg)	100 (80–120)	100 (80–118)	100 (80–120)	0.859
Crystalloid (mL)	600 (475–800)	600 (400–800)	700 (475–805)	0.650
Estimated blood loss (mL)	100 (75–200)	100 (50–125)	100 (100–200)	0.112
Urine output (mL)	100 (55–140)	90 (50–263)	100 (58–253)	0.811
Duration of operation (min)	125 (105–180)	125 (95–150)	125 (95–170)	0.892
Duration of OLV (min)	115 (85–133)	105 (85–125)	100 (90–130)	0.821
Duration of anesthesia (min)	175 (150–228)	170 (150–200)	175 (145–223)	0.907

Data are presented as numbers of patients or medians (interquartile ranges). There are no significant differences observed among the three groups. *LV* low tidal volume, *MV* medium tidal volume, *HV* high tidal volume, *ASA* American Society of Anesthesiologists, *FEV*_*1*_ forced expiratory volume in 1 s, *FVC* forced vital capacity, *VATS* video-assisted thoracic surgery, *OLV* one-lung ventilation

### Primary outcomes

**Fig. 2:**
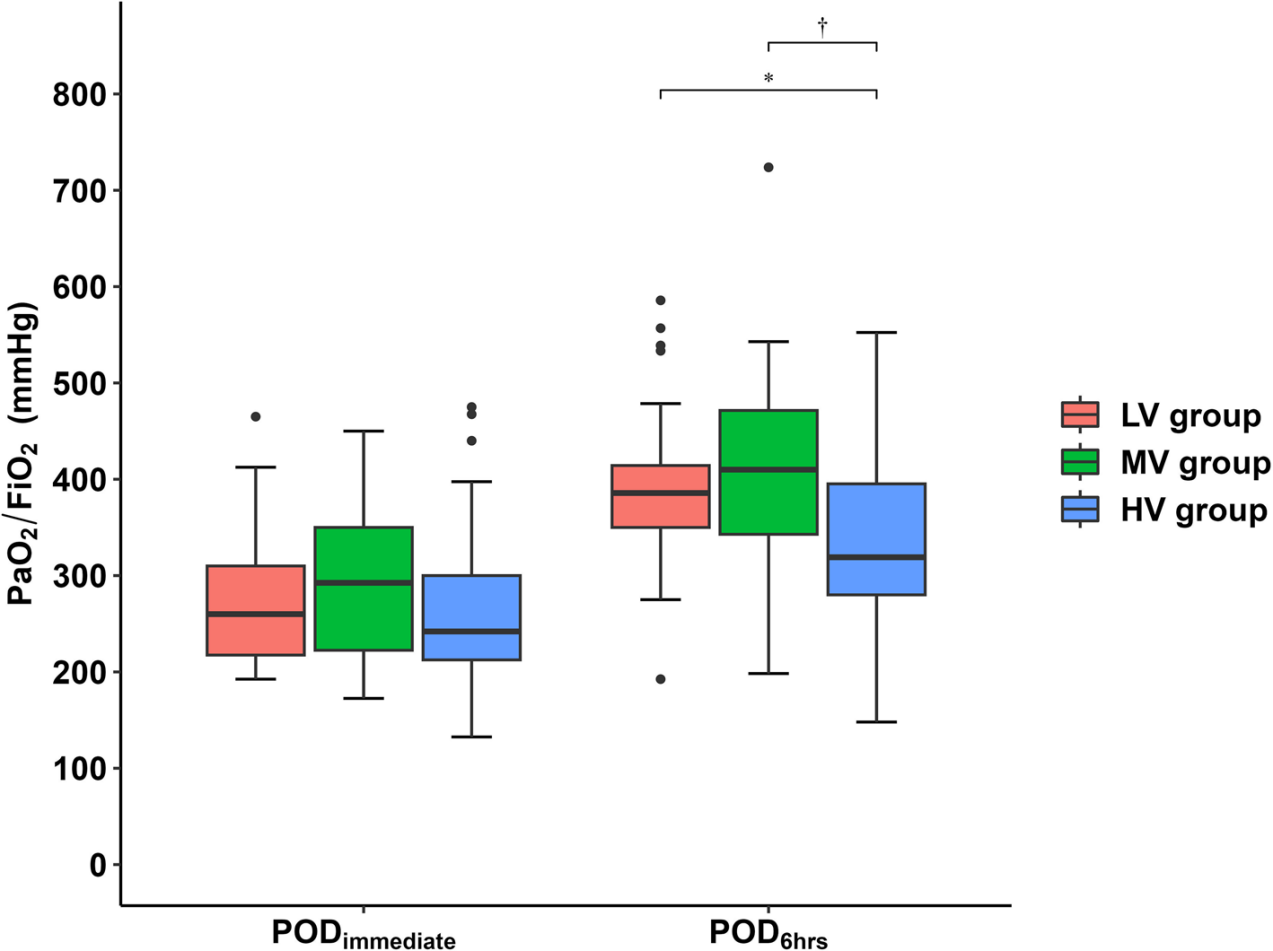
PaO_2_/FiO_2_ ratio measured immediately after arrival at the postanesthetic care unit or surgical intensive care unit and 6 h after surgery. The MV group shows the highest PaO_2_/FiO_2_ value among the three groups at 6 h after surgery (*P* = 0.010, Kruskal–Wallis *H* test). ^*^P = 0.042, LV vs*.* HV groups, adjusted by Bonferroni’s correction for post hoc analysis. ^†^*P* = 0.016, MV vs. HV groups, adjusted by Bonferroni’s correction for post hoc analysis. *LV* low tidal volume, *MV* medium tidal volume, *HV* high tidal volume, *PaO*_*2*_ arterial tension of oxygen, *FiO*_*2*_ inspired oxygen fraction

Among the three groups, the MV group showed the highest PaO_2_/FiO_2_ ratio at 6 h after the surgery (*P* = 0.010) (Fig. 2). The median (interquartile range) values for the LV, MV, and HV groups were 385 (341–416), 410 (340–471), and 319 (273–396), respectively. At this time point, the LV group presented the lowest incidence of PaO_2_/FiO_2_ < 300 mmHg among the LV, MV, and HV groups (2 vs. 3 vs. 11, respectively; *P* = 0.004). Since there was no among-group difference in the applied FiO_2_ postoperatively (LV group median [interquartile range], 0.28 [0.21–0.36]; MV group, 0.21 [0.21–0.34]; HV group, 0.21 [0.21–0.32]; *P* = 0.656), applying V_T_ of 4 mL/kg was not inferior to applying V_T_ of 8 mL/kg, albeit it allowed better oxygenation (*P* = 0.025).

The radiological findings of ventilator-induced acute lung lesions showed no differences among the three groups (Table 2). Patients in the HV group showed the highest incidence of lung lesions; however, the difference was not statistically significant (11/29 [37.9%] vs. 8/29 [27.6%] vs. 13/29 [44.8%]; *P* = 0.391).

**Table 2:** Lung lesions developed at 3 postoperative days

	LV group (n = 29)	MV group (n = 29)	HV group (n = 29)
Lung infiltration	7	6	10
(ipsilateral/contralateral/bilateral)	(3/3/1)	(1/5/0)	(8/2/0)
Atelectasis	5	3	6
(ipsilateral/contralateral/bilateral)	(2/1/2)	½/0	2/4/0
Lung infiltration + atelectasis	11	8	13

Data are represented as the number of patients with lung lesions. *LV* low tidal volume, *MV* medium tidal volume, *HV* high tidal volume

**Fig. 3:**
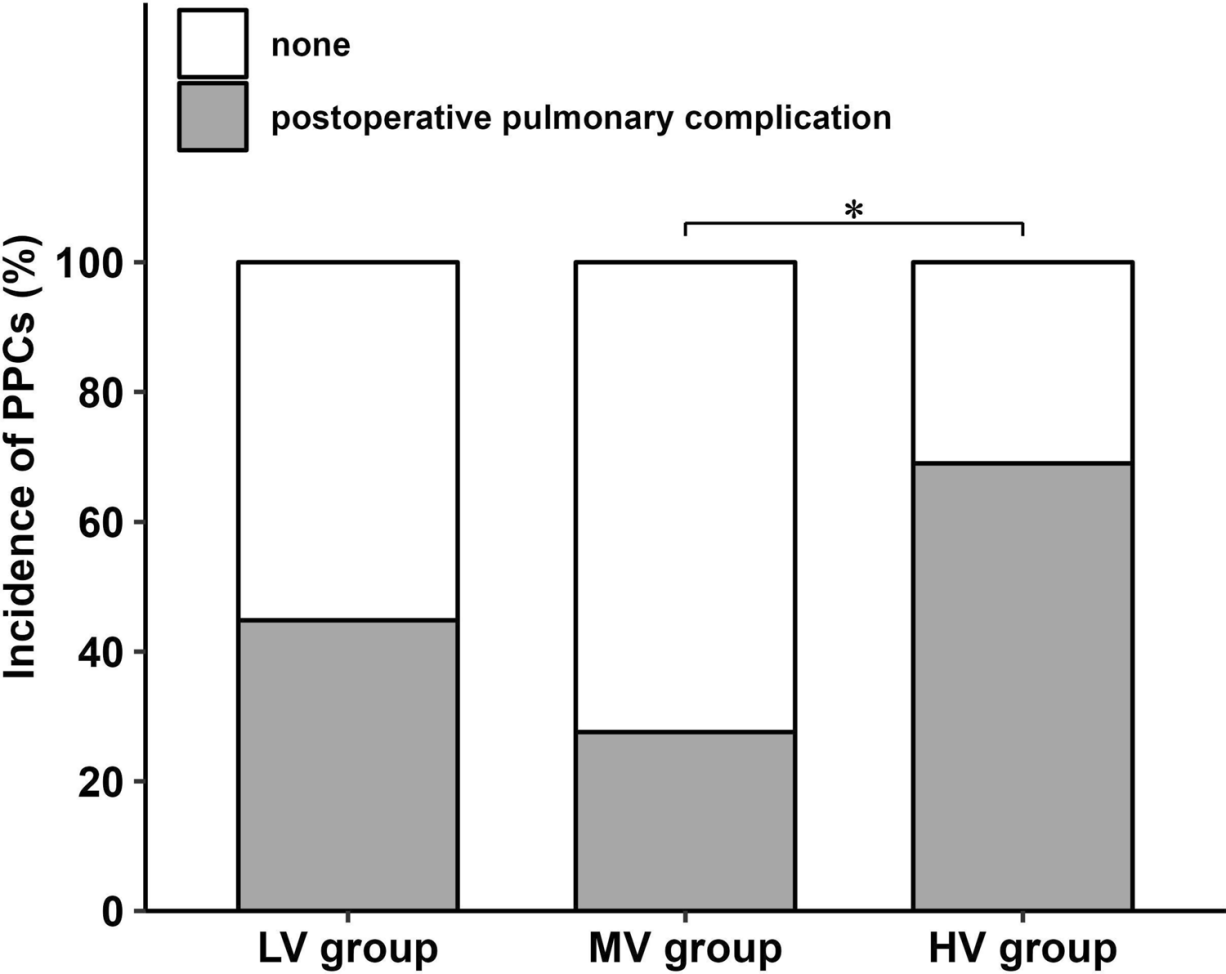
The incidence of immediate postoperative pulmonary complications. The incidences of postoperative pulmonary complications are represented as values. The MV group shows the lowest incidence among the three groups (*P* = 0.007, chi-squared test). LV, low tidal volume; MV, medium tidal volume; HV, high tidal volume. ^*^Odds ratio (95% confidence interval) 5.833 (1.88–18.099), *P* = 0.002, MV vs. HV group

Among the 87 patients, 41 (47.1%) developed immediate PPCs within 72 h of lung resection. The MV group showed the lowest incidence of PPCs among the three groups (13/29 [44.8%] vs. 8/29 [27.6%] vs. 20/29 [69.0%], *P* = 0.007) (Fig. 3). Specifically, the MV group showed a significantly lower incidence of PPCs compared with the HV group (odds ratio [95% confidence interval], 5.833 [1.88–18.099], *P* = 0.002). There were no significant differences in the incidence of PPCs between the LV and MV/HV groups (*P* = 0.823 and *P* = 0.335, respectively).

### Secondary outcomes

The intraoperative heart rate, mean blood pressure, and SpO_2_ showed no among-group differences at each measurement time point. Table 3 shows the intraoperative ventilatory variables. During OLV, there were significant among-group differences in P_mean_, P_plat_, PIP, and calculated P_drive_. Compared with the other groups, the HV group showed higher values, which reflected increased airway pressure, probably due to the high V_T_.

**Table 3:** Intraoperative ventilatory variables

Variables		LV group (n = 29)	MV group (n = 29)	HV group (n = 29)	*P* value
P_mean_					
	T_TLV_baseline_	8 (8–9)	8 (8–9)	8 (8–9)	0.749
	T_OLV_20min_	8 (8–9)^*^	9 (8–10)^**^	10 (9–10)	< 0.001
	T_OLV_40min_	9 (8–9)^*^	9 (8–10)^**^	10 (9–10)	0.001
	T_OLV_60min_	9 (8–9)^*^	9 (8–10)	10 (9–10)	0.013
	T_TLV_20min_	9 (8–10)	9 (8–10)	9 (9–10)	0.526
P_plat_					
	T_TLV_baseline_	16 (14–18)	17 (14–18)	17 (15–19)	0.817
	T_OLV_20min_	15 (14–17)^*^	17 (15–19)^**^	20 (18–21)	< 0.001
	T_OLV_40min_	16 (14–18)^*^	18 (16–19)^**^	20 (18–22)	< 0.001
	T_OLV_60min_	16 (15–19)^*^	18 (15–19)^**^	20 (18–22)	< 0.001
	T_TLV_20min_	22 (19–26)	20 (17–22)	20 (19–22)	0.139
PIP					
	T_TLV_baseline_	18 (16–20)	18 (16–19)	18 (16–20)	0.884
	T_OLV_20min_	17 (16–20)^*^	19 (16–22)^**^	22 (21–24)	< 0.001
	T_OLV_40min_	19 (16–21)^*^	20 (17–24)^**^	22 (20–26)	< 0.001
	T_OLV_60min_	19 (17–20)^*^	20 (17–24)	23 (21–24)	< 0.001
	T_TLV_20min_	23 (20–28)^***^	20 (18–22)	21 (21–24)	0.036
P_drive_					
	T_TLV_baseline_	11 (9–13)	12 (9–13)	12 (10–13)	0.918
	T_OLV_20min_	10 (9–12)^*^	12 (10–14)^**^	15 (13–16)	< 0.001
	T_OLV_40min_	11 (9–13)^*^	13 (11–15)^**^	15 (13–17)	< 0.001
	T_OLV_60min_	11 (10–14)^*^	13 (11–15)^**^	15 (13–17)	< 0.001
	T_TLV_20min_	17 (14–21)	15 (12–17)	15 (14–17)	0.116
FiO_2_					
	T_TLV_baseline_	1.00 (1.00–1.00)	1.00 (1.00–1.00)	1.00 (1.00–1.00)	0.368
	T_OLV_20min_ T_OLV_40min_ T_OLV_60min_ T_TLV_20min_	0.50 (0.50–0.55) 0.50 (0.50–0.60) 0.55 (0.50–0.80) 0.40 (0.40–0.40)	0.50 (0.50–0.60) 0.50 (0.50–0.57) 0.50 (0.50–0.60) 0.40 (0.40–0.40)	0.50 (0.50–0.55) 0.50 (0.50–0.60) 0.60 (0.50–0.80) 0.40 (0.40–0.40)	0.873 0.312 0.308 0.227

Data are expressed as medians (interquartile ranges). *P* values are computed using the Kruskal–Wallis *H* test

The HV group shows the highest value of ventilatory variables during OLV

^*^P < 0.05, LV vs*.* HV groups, adjusted by Bonferroni correction for post hoc analysis

^**^P < 0.05, MV vs. HV group, adjusted by Bonferroni’s correction for post hoc analysis

^***^P < 0.05, LV vs. MV group, adjusted by Bonferroni’s correction for post hoc analysis

*LV* low tidal volume, *MV* medium tidal volume, *HV* high tidal volume, *PIP* peak inspiratory pressure, *OLV* one-lung ventilation, *TLV* two-lung ventilation, *P*_*mean*_ mean airway pressure, *P*_*plat*_ airway plateau pressure, *PIP* peak inspiratory pressure, *P*_*drive*_ airway driving pressure, *T*_*TLV_baseline*_ baseline time point after induction of anesthesia, *T*_*OLV_20min*_ 20 min after the initiation of OLV, *T*_*OLV_40min*_ 40 min after the initiation of OLV, *T*_*OLV_60min*_ 60 min after the initiation of OLV, *T*_*TLV_20min*_ 20 min after the restoration of TLV

**Fig. 4:**
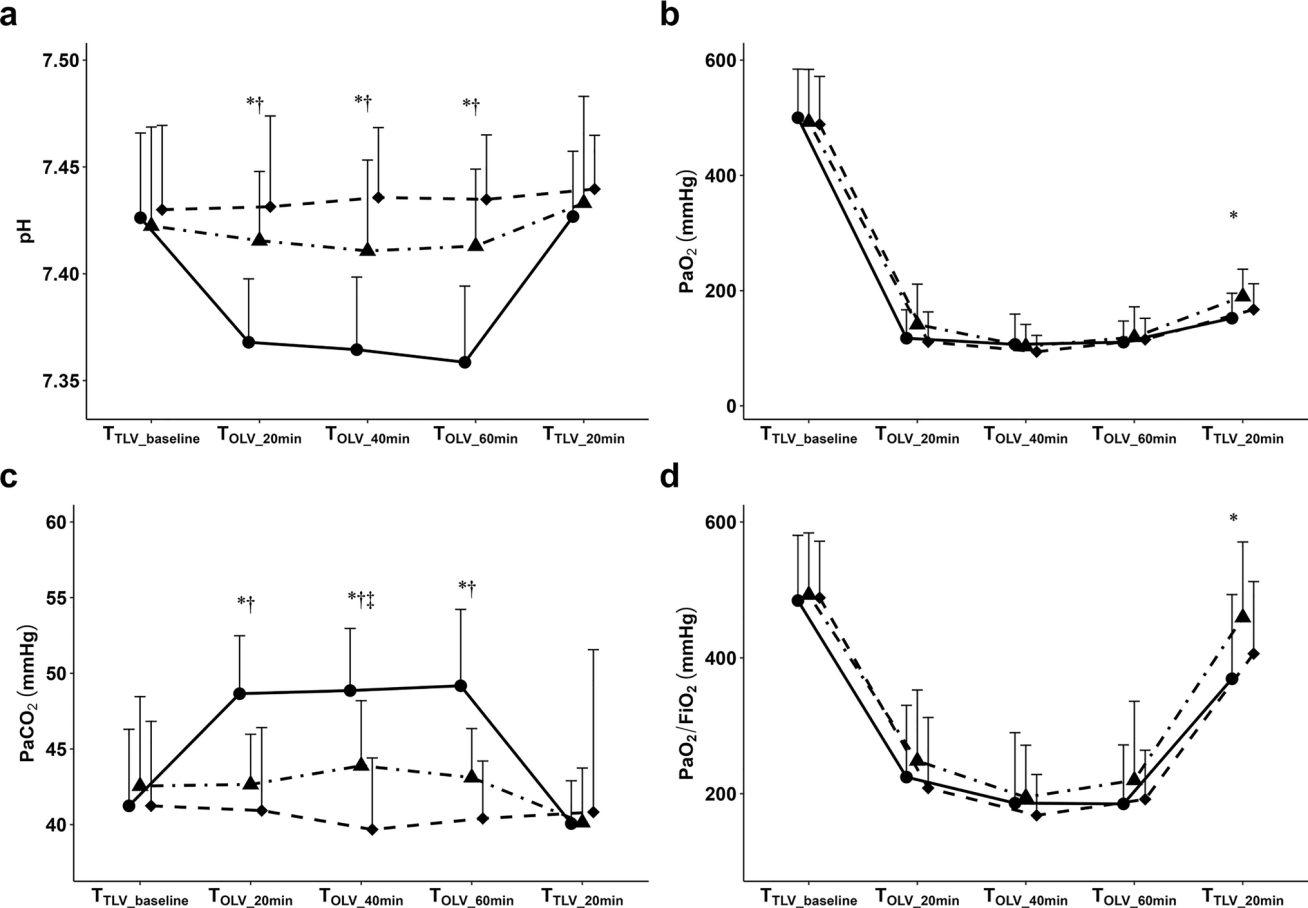
Intraoperative arterial pH (**a**), PaO_2_ (**b**), PaCO_2_ (**c**), and PaO_2_/FiO_2_ ratio (**d**) by arterial blood gas analysis at each measurement time point. LV group (●), MV group (▲), HV group (◆). Values are presented as medians and third quartiles. *P* values are computed using the Kruskal–Wallis *H* test. ^*^P < 0.05, LV vs. MV groups, adjusted by Bonferroni’s correction for post hoc analysis. ^†^*P* < 0.05, LV vs. HV groups, adjusted by Bonferroni’s correction for post hoc analysis. ^‡^*P* < 0.05, MV vs. HV groups, adjusted by Bonferroni’s correction for post hoc analysis. *LV* low tidal volume, *MV* medium tidal volume, *HV* high tidal volume, *pH* arterial pH, *PaO*_*2*_ arterial tension of oxygen, *PaCO*_*2*_ arterial tension of carbon dioxide, *FiO*_*2*_ inspired oxygen fraction, *TLV* two-lung ventilation, *T*_*TLV_baseline*_ baseline time point after the induction of anesthesia, *T*_*OLV_20min*_ 20 min after the initiation of OLV, *T*_*OLV_40min*_ 40 min after the initiation of OLV, *T*_*OLV_60min*_ 60 min after the initiation of OLV, *T*_*TLV_20min*_ 20 min after the restoration of TLV

There were significant among-group differences in arterial pH and PaCO_2_ during OLV (Fig. 4). The LV group showed hypercapnia during OLV along with a relatively low arterial pH, which was clinically acceptable. There were no significant among-group differences in terms of FiO_2_ or PaO_2_, which remained stable throughout OLV.

There were no significant among-group differences in the postoperative pain score (median (interquartile range), 7 (5–8) vs. 7 (5–8) vs. 6 (5–7), *P* = 0.304, respectively), mean length of stay in the PACU or SICU, and hospitalization (*P* = 0.605, *P* = 0.095, and *P* = 0.683, respectively). The mean length of hospital stay was 7 days in all groups. No patients presented postoperative pneumonia or acute respiratory distress syndrome requiring additional treatment. The mortality rate in the patients who received OLV within the first 30 postoperative days was 0%.

## Discussion

To the best of our knowledge, this is the first study to explore an optimal protective ventilation strategy for OLV during lung resection surgery, from the perspective of postoperative lung oxygenation. We found that ventilation at a V_T_ of 6 mL/kg showed the highest PaO_2_/FiO_2_ value at 6 h after surgery and the lowest incidence of immediate PPCs than those at a V_T_ of 4 mL/kg and 8 mL/kg when all ventilations were conducted at a PEEP of 5 cmH_2_O.

Oxygenation and lung protection are the major concerns for OLV in thoracic surgery [18–20]. Since protective ventilation strategies differ in terms of various ventilatory variables, a standard strategy for protective OLV remains to be established.

Several clinical studies have suggested that a V_T_ of 4–8 mL/kg with a certain amount of PEEP is advantageous in preventing PPCs after lung resection in adult and infant patients for intraoperative oxygenation and postoperative progress of lung consolidation [21–29]. Another randomized controlled physiological study revealed that a V_T_ of 4 mL/kg during OLV reduced extravascular lung water content index, which is a useful marker of pulmonary complication [17]. In addition, an animal study showed decreased release of proinflammatory cytokines when applying a lung-protective ventilation strategy, which was beneficial for postoperative lung inflammatory responses [30].

Some studies have reported no significant differences in clinical variables between protective and conventional ventilation strategies. However, those were mostly observational cohort studies or randomized controlled trials conducted in humans without regulating other factors affecting protective ventilation strategies, including PEEP, FiO_2_, or alveolar recruitment [2, 31–34].

Our study is the first randomized controlled trial to compare postoperative PaO2/FiO2 ratio following three protective ventilation strategies during OLV for lung resection surgery. Given that a lack of PEEP would worsen oxygenation and the shunt fraction during OLV, we applied a PEEP of 5 cmH_2_O in all three study groups. Notably, a V_T_ of 6 mL/kg during OLV was more beneficial in preventing PPCs than the other V_T_ values.

This result may be attributed to the combination of the following variables assessed: postoperative PaO_2_/FiO_2_ and newly developed lung lesions. PaO_2_/FiO_2_ reflects tissue oxygenation and damage to the lung vessels and alveoli; therefore, it is commonly used as a diagnostic criterion for ALI [35–37]. Although the mean PaO_2_, applied FiO_2_, or PaO_2_/FiO_2_ ratio during OLV did not show significant among-group differences, the LV and MV groups showed significantly higher PaO_2_/FiO_2_ ratios than the HV group at 6 h after surgery. Furthermore, the LV group had the lowest incidence of PaO_2_/FiO_2_ < 300 mmHg. This implies that a low V_T_ does not adversely affect deoxygenation or induce lung injury if adequate PEEP is applied simultaneously. The application of PEEP minimizes the alveolar collapse of the ventilated lung and atelectrauma, which contribute to lung injury during mechanical ventilation [38, 39]. This may be associated with improved oxygenation and reduced lung injury in the LV and MV groups.

Moreover, at the time of OLV initiation, the LV group exhibited higher PaCO_2_ levels than the other groups. Given that we attempted to adjust the ventilator respiratory rate mostly according to EtCO2 to maintain the PaCO2 at 35–45 mmHg since it is more intuitive, PaCO_2_ levels showed deviated results in the range of 44–52 mmHg during OLV in the LV group (Fig. 4). Since a moderate degree of hypercapnia potentiates the hypoxic pulmonary vasoconstriction response during OLV, CO_2_ retention improved oxygenation in the LV group compared with that in the HV group, which may have contributed to the favorable outcomes in the incidence of PPCs [40].

Hyperinflation, hyperoxia, and hyperperfusion in the dependent lung at high V_T_ during OLV can cause exaggerated inflammatory and oxidative damage, which subsequently leads to ALI [41]. Although a high V_T_ during mechanical ventilation is correlated with a low incidence of atelectasis, high ventilation pressure is associated with lung injury. In our study, intraoperative PIP, P_mean_, P_plat_, and calculated P_drive_ were significantly higher in the HV group, than in the other groups, as expected, which contributed to the HV group having the highest incidence of lung infiltration according to radiological findings (11 vs. 8 vs. 13).

Although it is rational to avoid high V_T_ and FiO_2_ as well as to apply PEEP with alveolar recruitment, an optimal strategy remains to be established. Several anesthesiologists remain hesitant about minimizing V_T_ and FiO_2_ in the clinical application of a protective ventilation strategy during OLV, which is strongly associated with hypoxemia. In our study, applying a V_T_ of 4 mL/kg resulted in a similar incidence of PaO_2_/FiO_2_ < 300 mmHg to that when applying a V_T_ of 6 mL/kg, albeit with a higher incidence of atelectasis in the radiological findings. In addition, a V_T_ of 8 mL/kg resulted in a high incidence of PaO_2_/FiO_2_ < 300 mmHg, which was related to postoperative lung infiltration, as previously reported [8, 22, 24, 28]. Therefore, our study will help determine the appropriate V_T_ in a ventilator setting as a protective ventilation strategy during OLV.

This study has several limitations. First, chest radiological evaluations were focused on newly developed lung infiltrations or atelectasis. Since definitive features of ALI include inflammation detected on chest radiographs, we defined PPCs to include these radiological findings. This probably contributed to the relatively high incidence of PPCs in our study compared to previous studies. However, early recognition and appropriate management are crucial for improving postoperative outcomes as well as reducing the risk of disease progression in patients undergoing OLV for thoracic surgery. Therefore, the primary outcome of our study, which may be considered broad, can be clinically meaningful. The measurement of biomarkers of pulmonary inflammation would strengthen our results by directly assessing lung damage. Second, both open thoracotomy and video-assisted thoracic surgery were included in the study. However, we applied epidural catheterization for postoperative pain control in patients who underwent open thoracotomy to reduce surgical stress, including postoperative pain, which could lead to impaired oxygenation due to pain-induced shallow breathing in immediate postoperative period. Accordingly, there was no significant among-group difference in postoperative pain. Third, the small sample size may limit the generalizability of our results. Accordingly, large-scale studies are warranted to investigate differences in the PaO_2_/FiO_2_ ratio and the incidence of newly developed lung lesions.

## Conclusions

Our findings suggest that a protective ventilation strategy at a V_T_ of 6 mL/kg with PEEP of 5 cmH_2_O may contribute to a higher postoperative PaO_2_/FiO_2_ ratio with a reduced incidence of immediate PPCs in lung resection surgery. Further studies are required to confirm the clinical importance of a specific protective ventilation strategy with long-term benefits in thoracic surgery.

## Data Availability

The datasets analyzed in this study are available from the corresponding author upon request.

## References

[1] Serpa Neto A, Hemmes SN, Barbas CS, Beiderlinden M, Fernandez-Bustamante A, Futier E, Hollmann MW, Jaber S, Kozian A, Licker M, Lin WQ, Moine P, Scavonetto F, Schilling T, Selmo G, Severgnini P, Sprung J, Treschan T, Unzueta C, Weingarten TN, Wolthuis EK, Wrigge H, Gama de Abreu M, Pelosi P, Schultz MJ, Network PROVE, investigators (2014) Incidence of mortality and morbidity related to postoperative lung injury in patients who have undergone abdominal or thoracic surgery: a systematic review and meta-analysis. Lancet Respir Med 2:1007–1015 25466352 10.1016/S2213-2600(14)70228-0

[2] Colquhoun DA, Leis AM, Shanks AM, Mathis MR, Naik BI, Durieux ME, Kheterpal S, Pace NL, Popescu WM, Schonberger RB, Kozower BD, Walters DM, Blasberg JD, Chang AC, Aziz MF, Harukuni I, Tieu BH, Blank RS (2021) A lower tidal volume regimen during one-lung ventilation for lung resection surgery is not associated with reduced postoperative pulmonary complications. Anesthesiology 134:562–576 33635945 10.1097/ALN.0000000000003729 PMC8274370

[3] Amato MB, Barbas CS, Medeiros DM, Magaldi RB, Schettino GP, Lorenzi-Filho G, Kairalla RA, Deheinzelin D, Munoz C, Oliveira R, Takagaki TY, Carvalho CR (1998) Effect of a protective-ventilation strategy on mortality in the acute respiratory distress syndrome. N Engl J Med 338:347–354 9449727 10.1056/NEJM199802053380602

[4] Lohser J (2008) Evidence-based management of one-lung ventilation. Anesthesiol Clin 26:241–272 18456211 10.1016/j.anclin.2008.01.011

[5] Camilo LM, Ávila MB, Cruz LF, Ribeiro GC, Spieth PM, Reske AA, Amato M, Giannella-Neto A, Zin WA, Carvalho AR (2014) Positive end-expiratory pressure and variable ventilation in lung-healthy rats under general anesthesia. PLoS ONE 9:e110817 25383882 10.1371/journal.pone.0110817 PMC4226529

[6] De Prost N, Dreyfuss D (2012) How to prevent ventilator-induced lung injury? Minerva Anestesiol 78:1054–1066 22772855

[7] Serpa Neto A, Cardoso SO, Manetta JA, Pereira VG, Espósito DC, Pasqualucci Mde O, Damasceno MC, Schultz MJ (2012) Association between use of lung-protective ventilation with lower tidal volumes and clinical outcomes among patients without acute respiratory distress syndrome: a meta-analysis. JAMA 308:1651–1659 23093163 10.1001/jama.2012.13730

[8] Kozian A, Schilling T, Schütze H, Senturk M, Hachenberg T, Hedenstierna G (2011) Ventilatory protective strategies during thoracic surgery: effects of alveolar recruitment maneuver and low-tidal volume ventilation on lung density distribution. Anesthesiology 114:1025–1035 21436678 10.1097/ALN.0b013e3182164356

[9] Della Rocca G, Coccia C (2011) Ventilatory management of one-lung ventilation. Minerva Anestesiol 77:534–536 21540809

[10] Fu Y, Zhang YW, Gao J, Fu HM, Si L, Gao YT (2012) Effects of lung-protective ventilation strategy on lung aeration loss and postoperative pulmonary complications in moderate-risk patients undergoing abdominal surgery. Minerva Anestesiol 87:655–662 10.23736/S0375-9393.20.14951-4 33325216

[11] Schmidt AP, Marques AJ, Reinstein AR, Bevilacqua Filho CT, Carmona MJC, Auler JOC Jr, Felix EA, Andrade CF (2020) Effects of protective mechanical ventilation during general anesthesia in patients undergoing peripheral vascular surgery: a randomized controlled trial. J Clin Anesth 61:109656 31784303 10.1016/j.jclinane.2019.109656

[12] Costa Leme A, Hajjar LA, Volpe MS, Fukushima JT, De Santis Santiago RR, Osawa EA, Pinheiro de Almeida J, Gerent AM, Franco RA, Zanetti Feltrim MI, Nozawa E, de Moraes Coimbra VR, de Moraes IR, Hashizume CS, Kalil Filho R, Auler JO Jr, Jatene FB, Gomes Galas FR, Amato MB (2017) Effect of intensive vs moderate alveolar recruitment strategies added to lung-protective ventilation on postoperative pulmonary complications: a randomized clinical trial. JAMA 317:1422–1432 28322416 10.1001/jama.2017.2297

[13] Yang D, Grant MC, Stone A, Wu CL, Wick EC (2016) A Meta-analysis of Intraoperative Ventilation Strategies to Prevent Pulmonary Complications: Is Low Tidal Volume Alone Sufficient to Protect Healthy Lungs? Ann Surg 263:881–887 26720429 10.1097/SLA.0000000000001443

[14] El Tahan MR, Samara E, Marczin N, Landoni G, Pasin L (2023) Impact of lower tidal volumes during one-lung ventilation: a 2022 update of the meta-analysis of randomized controlled trials. J Cardiothorac Vasc Anesth 37:1983–1992 37225546 10.1053/j.jvca.2023.04.018

[15] Levin MA, McCormick PJ, Lin HM, Hosseinian L, Fischer GW (2014) Low intraoperative tidal volume ventilation with minimal PEEP is associated with increased mortality. Br J Anaesth 113:97–108 24623057 10.1093/bja/aeu054 PMC9585620

[16] Uhlig C, Neto AS, van der Woude M, Kiss T, Wittenstein J, Shelley B, Scholes H, Hiesmayr M, Vidal Melo MF, Sances D, Coskunfirat N, Pelosi P, Schultz M, Gama de Abreu M (2020) Intraoperative mechanical ventilation practice in thoracic surgery patients and its association with postoperative pulmonary complications: results of a multicenter prospective observational study. BMC Anesthesiol 20:179 32698775 10.1186/s12871-020-01098-4 PMC7373838

[17] Qutub H, El-Tahan MR, Mowafi HA, El Ghoneimy YF, Regal MA, Al Saflan AA (2014) Effect of tidal volume on extravascular lung water content during one-lung ventilation for video-assisted thoracoscopic surgery: a randomised, controlled trial. Eur J Anaesthesiol 31:466–473 24690891 10.1097/EJA.0000000000000072

[18] Heerdt PM, Stowe DF (2017) Single-lung ventilation and oxidative stress: a different perspective on a common practice. Curr Opin Anaesthesiol 30:42–49 27783023 10.1097/ACO.0000000000000410

[19] Lohser J, Slinger P (2015) Lung injury after one-lung ventilation: a review of the pathophysiologic mechanisms affecting the ventilated and the collapsed lung. Anesth Analg 121:302–318 26197368 10.1213/ANE.0000000000000808

[20] Şentürk M, Slinger P, Cohen E (2015) Intraoperative mechanical ventilation strategies for one-lung ventilation. Best Pract Res Clin Anaesthesiol 29:357–369 26643100 10.1016/j.bpa.2015.08.001

[21] Feng Y, Wang J, Zhang Y, Wang S (2016) One-lung ventilation with additional ipsilateral ventilation of low tidal volume and high frequency in lung lobectomy. Med Sci Monit 22:1589–1592 27166086 10.12659/MSM.895294 PMC4913818

[22] Fernández-Pérez ER, Keegan MT, Brown DR, Hubmayr RD, Gajic O (2006) Intraoperative tidal volume as a risk factor for respiratory failure after pneumonectomy. Anesthesiology 105:14–18 16809989 10.1097/00000542-200607000-00007

[23] Michelet P, D’Journo XB, Roch A, Doddoli C, Marin V, Papazian L, Decamps I, Bregeon F, Thomas P, Auffray JP (2006) Protective ventilation influences systemic inflammation after esophagectomy: a randomized controlled study. Anesthesiology 105:911–919 17065884 10.1097/00000542-200611000-00011

[24] Yang M, Ahn HJ, Kim K, Kim JA, Yi CA, Kim MJ, Kim HJ (2011) Does a protective ventilation strategy reduce the risk of pulmonary complications after lung cancer surgery?: a randomized controlled trial. Chest 139:530–537 20829341 10.1378/chest.09-2293

[25] Kidane B, Choi S, Fortin D, O’Hare T, Nicolaou G, Badner NH, Inculet RI, Slinger P, Malthaner RA (2018) Use of lung-protective strategies during one-lung ventilation surgery: a multi-institutional survey. Ann Transl Med 6:269 30094255 10.21037/atm.2018.06.02 PMC6064789

[26] Marret E, Cinotti R, Berard L, Piriou V, Jobard J, Barrucand B, Radu D, Amziane S, Bachir Bouiadjra M (2018) Protective ventilation during anaesthesia reduces major postoperative complications after lung cancer surgery: a double-blind randomised controlled trial. Eur J Anaesthesiol 35:727–735 29561278 10.1097/EJA.0000000000000804

[27] Licker M, Diaper J, Villiger Y, Spiliopoulos A, Licker V, Robert J, Tschopp JM (2009) Impact of intraoperative lung-protective interventions in patients undergoing lung cancer surgery. Crit Care 13:R41 19317902 10.1186/cc7762 PMC2689485

[28] Lee JH, Bae JI, Jang YE, Kim EH, Kim HS, Kim JT (2019) Lung protective ventilation during pulmonary resection in children: a prospective, single-centre, randomised controlled trial. Br J Anaesth 122:692–701 30916035 10.1016/j.bja.2019.02.013

[29] Santschi M, Randolph AG, Rimensberger PC, Jouvet P (2013) Mechanical ventilation strategies in children with acute lung injury: a survey on stated practice pattern*. Pediatr Crit Care Med 14:e332–e337 23842587 10.1097/PCC.0b013e31828a89a2

[30] Theroux MC, Fisher AO, Horner LM, Rodriguez ME, Costarino AT, Miller TL, Shaffer TH (2010) Protective ventilation to reduce inflammatory injury from one lung ventilation in a piglet model. Paediatr Anaesth 20:356–364 19919624 10.1111/j.1460-9592.2009.03195.x

[31] Maslow AD, Stafford TS, Davignon KR, Ng T (2013) A randomized comparison of different ventilator strategies during thoracotomy for pulmonary resection. J Thorac Cardiovasc Surg 146:38–44 23380515 10.1016/j.jtcvs.2013.01.021

[32] Ahn HJ, Kim JA, Yang M, Shim WS, Park KJ, Lee JJ (2012) Comparison between conventional and protective one-lung ventilation for ventilator-assisted thoracic surgery. Anaesth Intensive Care 40:780–788 22934859 10.1177/0310057X1204000505

[33] Amar D, Zhang H, Pedoto A, Desiderio DP, Shi W, Tan KS (2017) Protective lung ventilation and morbidity after pulmonary resection: a propensity score-matched analysis. Anesth Analg 125:190–199 28598916 10.1213/ANE.0000000000002151

[34] El Tahan MR, Pasin L, Marczin N, Landoni G (2017) Impact of low tidal volumes during one-lung ventilation. a meta-analysis of randomized controlled trials. J Cardiothorac Vasc Anesth 31:1767–1773 28843606 10.1053/j.jvca.2017.06.015

[35] Licker M, de Perrot M, Spiliopoulos A, Robert J, Diaper J, Chevalley C, Tschopp JM (2003) Risk factors for acute lung injury after thoracic surgery for lung cancer. Anesth Analg 97:1558–1565 14633519 10.1213/01.ANE.0000087799.85495.8A

[36] Matthay MA (1999) Conference summary: acute lung injury. Chest 116:119s-s126 10424631 10.1378/chest.116.suppl_1.119s

[37] Talmor D, Sarge T, Malhotra A, O’Donnell CR, Ritz R, Lisbon A, Novack V, Loring SH (2008) Mechanical ventilation guided by esophageal pressure in acute lung injury. N Engl J Med 359:2095–2104 19001507 10.1056/NEJMoa0708638 PMC3969885

[38] Muscedere JG, Mullen JB, Gan K, Slutsky AS (1994) Tidal ventilation at low airway pressures can augment lung injury. Am J Respir Crit Care Med 149:1327–1334 8173774 10.1164/ajrccm.149.5.8173774

[39] Duggan M, McCaul CL, McNamara PJ, Engelberts D, Ackerley C, Kavanagh BP (2003) Atelectasis causes vascular leak and lethal right ventricular failure in uninjured rat lungs. Am J Respir Crit Care Med 167:1633–1640 12663325 10.1164/rccm.200210-1215OC

[40] Lang CJ, Barnett EK, Doyle IR (2006) Stretch and CO2 modulate the inflammatory response of alveolar macrophages through independent changes in metabolic activity. Cytokine 33:346–351 16713281 10.1016/j.cyto.2006.03.006

[41] Williams EA, Quinlan GJ, Goldstraw P, Gothard JW, Evans TW (1998) Postoperative lung injury and oxidative damage in patients undergoing pulmonary resection. Eur Respir J 11:1028–1034 9648951 10.1183/09031936.98.11051028

